# Liposomal formulation of Gp41 derivate with adjuvant MPLA: vaccine design, immunogenicity in animals and safety in humans

**DOI:** 10.1186/1742-4690-9-S2-P354

**Published:** 2012-09-13

**Authors:** D Katinger, A Wagner, I Luque, S Crespillo, F Conejero-Lara, M Roger, C Martin, N Mouz, S Mourao, A Farsang, F Notka, K Malcolm, N Bosquet, R Le Grand, C Moog, A Cope, R Shattock, DJ Lewis, R El Habib

**Affiliations:** 1 Polymun Scientific GmbH, Klosterneuburg, Austria; 2 Universidad de Granada, Granada, Spain; 3 PX'Therapeutics, Grenoble, France; 4 I.M. PROJET, Miribel, France; 5 National Food Chain Safety Office, Budapest, Hungary; 6 GeneArt AG / Life Technologies, Regensburg, Germany; 7 Queen's University of Belfast, Belfast, UK; 8 CEA, Fontenay-aux-Roses, France; 9 Université de Strasbourg, Strasbourg, France; 10 Imperial College, London, UK; 11 University of Surrey, Guildford, UK; 12 Sanofi Pasteur, Marcy l’Etoile, France

## Background

Gp41 of the HIV envelope, especially the MPER, contains highly conserved epitopes recognized by neutralizing monoclonal antibodies such as D5, 2F5 and 4E10. The correct presentation of the antigens is considered to be key for eliciting neutralizing immune response. The lipid bilayer of liposomes can mimic the virus surface and therefore provides the appropriate presentation of this membrane protein. In addition, liposomes can integrate the adjuvant monophosphoryl lipid A (MPLA).

## Methods

The gp41 derivate FPA2 was mutated to increase its solubility and modify its structure for exposure of neutralizing epitopes. The protein was expressed in E. coli. The purified protein was solubilized with detergent. Liposomes were prepared containing antigen FPA2 and MPLA, a Toll-like receptor 4 agonist, using a continuous ethanol crossflow injection technology. Immunogenicity and safety was determined in rabbits and macaques. The liposomes formulated in HEC gel were applied i.n. followed by two intramuscular boosts in healthy female volunteers in a phase I study of safety and immunogenicity.

## Results

Liposomes containing FPA2 and MPLA manufactured under GMP conditions were uniform and stable at 2-8°C. Immunogenicity was demonstrated in rabbits and monkey after mucosal (i.vag., i.n.) prime, followed by intramuscular boosts. Immune response was induced systemically and in mucosal surfaces. Preliminary data from the clinical phase 1 study in healthy volunteers demonstrated good safety with three nasal applications of 200 µg of the formulated protein in HEC gel and of two i.m. boosts.

## Conclusion

The liposomal formulation of the gp41 subunit of HIV envelope together with MPLA as adjuvant proved to be well tolerated in animals and human. Liposome-formulated FPA2 was able to induce binding antibodies measured by ELISA and neutralizing activity in both blood and vaginal secretions in animals. Immunogenicity testing in humans is ongoing.

